# A cross-sectional analysis of dietary selenium intake and type 2 diabetes risk in adults: insights from NHANES 2011–2016

**DOI:** 10.3389/fnut.2025.1583590

**Published:** 2025-05-09

**Authors:** Shenghui Ge, Baowen Yu, Xiao Yin, Deyue Kong, Yi Luo, Jianhua Ma, Wenqing Xia

**Affiliations:** Department of Endocrinology, Nanjing First Hospital, Nanjing Medical University, Nanjing, China

**Keywords:** dietary selenium intake, type 2 diabetes mellitus, obesity, cross-sectional study, NHANES

## Abstract

**Objective:**

While selenium exhibits antioxidant properties, its association with type 2 diabetes mellitus (T2DM) remains controversial. This study aimed to investigate the relationship between dietary selenium intake and T2DM risk in a nationally representative population.

**Methods:**

We conducted a cross-sectional analysis of 2,170 adults from the National Health and Nutrition Examination Survey (NHANES) 2011–2016. Using weighted multivariable logistic regression, we estimated adjusted ORs with 95% CIs across selenium intake quartiles. Restricted cubic splines with three knots (10th, 50th, and 90th percentiles) were employed to characterize non-linear associations. Additionally, stratified analyses were performed based on age, sex, ethnicity, BMI, smoking status, and drinking status.

**Results:**

A significant U-shaped relationship was observed between dietary selenium intake and T2DM risk (*p* for non-linearity = 0.042), indicating increased risk at both low and high intake extremes. In obese individuals (BMI ≥ 30 kg/m^2^), higher selenium intake was inversely associated with the risk of T2DM (*p*_trend_ = 0.016), suggesting a potential protective role in populations with elevated oxidative stress. No significant associations were found for supplemental or total selenium intake.

**Conclusion:**

Both insufficient and excessive dietary selenium intake may elevate the risk of T2DM, with an optimal range identified through non-linear modeling. Targeted selenium recommendations for obese individuals could mitigate diabetes risk, though longitudinal studies are needed to confirm causality. These findings highlight the importance of personalized nutrition strategies in high-risk populations.

## Introduction

Diabetes mellitus affected 537 million adults globally in 2021, with projections indicating a 46% increase to 783 million by 2045 (1). The most common form is type 2 diabetes mellitus (T2DM), accounting for approximately 98% of all diabetes diagnoses (2, 3). This escalating epidemic highlights the urgent need to identify modifiable risk factors and implement timely, effective interventions to mitigate its impact.

Selenium, an essential trace element, is crucial for human health as it integrates into selenoproteins with strong anti-inflammatory and antioxidant effects (4, 5). These properties are especially important for T2DM individuals, who often face increased oxidative stress and systemic inflammation (6). Such conditions elevate the body’s demand for antioxidants, such as selenium, to counteract cellular damage and restore metabolic balance.

Despite its biological significance, the relationship between selenium and the risk of T2DM remains controversial. While many studies have reported a positive association between higher selenium levels and T2DM risk, others have found no significant association (7–11). These discrepancies may stem from differences in study populations, dietary selenium sources, or adjustments for confounding factors. Furthermore, while the association between dietary selenium intake and diabetes has been extensively studied, there remains a lack of research exploring the non-linear relationship in a large adult population. Additionally, it is unclear whether factors such as sex, ethnicity, body mass index (BMI), smoking status, drinking status, and especially serum selenium levels might modify this association. Addressing these gaps could provide novel strategies for early health management, particularly for individuals with obesity.

To address these gaps, this study examined the association between dietary selenium intake and the risk of T2DM using a nationally representative sample of U.S. adults, with a focus on identifying non-linear relationships and potential effect modifiers.

## Research design and methods

### Study population

This study utilized publicly accessible, anonymized data from the National Health and Nutrition Examination Survey (NHANES 2011–2016) administered by the Centers for Disease Control and Prevention (CDC). Ethical approval for NHANES data collection was granted by the National Center for Health Statistics (NCHS) Research Ethics Review Board, and all participants provided documented informed consent during their original enrollment in NHANES.

**Figure 1:**
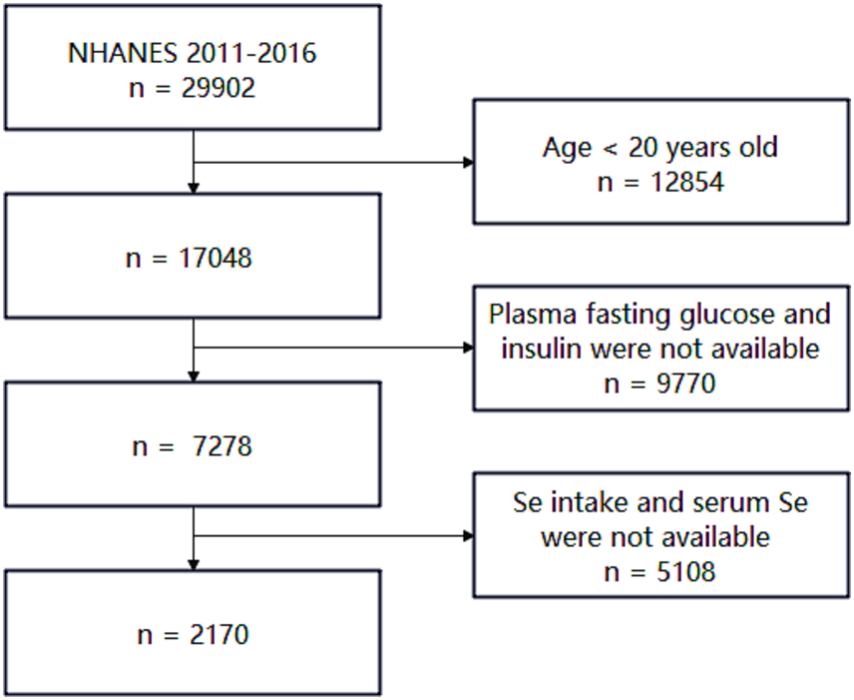
A flowchart of the individual selection from NHANES 2011–2016.

Inclusion criteria were as follows: (i) age ≥20 years, (ii) availability of plasma fasting glucose and plasma insulin data, and (iii) availability of dietary and plasma selenium data. Exclusion criteria included: (i) individuals under 20 years of age and (ii) individuals missing any of the aforementioned critical information. Details are shown in Figure 1. After screening, 2,170 participants were included in this study.

### Assessment of covariates

Covariates considered include several demographic characteristics: sex (male or female), age (years), ethnicity (Mexican American, other Hispanic, White non-Hispanic, Black non-Hispanic, or other ethnic/racial groups), BMI, family income (poverty income ratio, PIR), educational attainment (less than a high school education, high school graduate/GED, some high school, some college or associate’s degree, or college graduate or above), alcohol intake (non-drinker, 1 to <5 drinks/month, 5 to <10 drinks/month, or 10+ drinks/month), and smoking status (current, former, or never smoker). Never smokers were classified as those who had reported smoking <100 cigarettes during their lifetime. Current smokers were defined as those who had smoked >100 cigarettes over their lifetime and were actively smoking cigarettes at the time of the survey. Former smokers were defined as those who had smoked >100 cigarettes in their lifetime but had quit smoking before the time of the survey. Hypertension was defined as a self-reported doctor diagnosis of hypertension, systolic blood pressure ≥140 mmHg, or diastolic blood pressure ≥90 mmHg.

### Selenium in diet and supplements

In each NHANES cycle, participants provided detailed dietary intake information for two 24-h periods. The first dietary recall was conducted in person during the NHANES visit, while the second was collected by telephone 3–10 days later. For the analyses, the total estimated dietary selenium intake was computed by averaging the selenium intake over the two recall periods. If only data from the first day were available, that value was used instead of computing an average. Apart from dietary selenium intake, participants were queried about their supplement use during the same two 24-h periods. Selenium intake from supplements was also averaged over 2 days if data were available. The total selenium intake was then calculated as the sum of dietary selenium intake and supplement selenium intake.

Plasma fasting glucose, insulin, glycated hemoglobin A1c (HbA1c), albumin, total protein, and creatinine were measured at baseline. Strict procedures were applied during blood collection and analysis, and details were documented in the NHANES Laboratory/Medical Technologists Procedures Manual.

### T2DM diagnosis

T2DM was defined as self-reported physician diagnosis of diabetes, use of insulin or oral hypoglycemic medication, plasma fasting glucose ≥7.0 mmol/L (126 mg/dL), postprandial 2-h plasma glucose ≥11.1 mmol/L (200 mg/dL) from an oral glucose tolerance test, or HbA1c ≥ 6.5% (mmol/mol).

BMI was calculated as weight (in kilograms) divided by the square of height (in meters). Homeostasis model assessment of insulin resistance (HOMA-IR) was calculated by multiplying fasting glucose in mg/dL by fasting insulin in μU/mL and dividing by 22.5 (12). Statistical analysis, sample weights, strata, and primary sampling units were used to account for the complex survey design according to the NHANES analytic guidelines.

### Statistical analysis

All analyses incorporated sample weights, strata, and primary sampling units to produce accurate national estimates. Characteristics of the study population were expressed as medians (interquartile ranges) for continuous variables or percentages for categorical variables. Participants lacking critical biochemical indicators required for our primary analyses were excluded.

Logistic regression models were used to estimate the ORs and 95% CIs of dietary selenium intake and the risk of T2DM according to quartiles of dietary selenium intake. To investigate dose–response associations between selenium intake and the risk of T2DM, we used a restricted cubic spline regression model with three knots at the 10th, 50th, and 90th percentiles of the selenium intake distribution. We also investigated the association between selenium supplements and total selenium intake with the risk of T2DM.

We fitted three statistical models. Model 1 was adjusted for age (continuous), sex (male or female), and ethnicity (White non-Hispanic, Black non-Hispanic, Mexican American, other/multiracial, or other Hispanic). Model 2 was further adjusted for PIR (continuous), education level (college graduate or above, high school graduate/GED, less than high school, or some college or AA), BMI (continuous), smoking status (current smoker, former smoker, or never smoker), and drinking status (1–5 drinks/month, 5–10 drinks/month, 10+ drinks/month, or non-drinker). Model 3 was further adjusted for albumin (continuous), serum selenium (continuous), and serum creatinine (continuous).

Stratified analyses were conducted by age (20–39 years, 40–59 years, or 60+ years), sex (male or female), ethnicity (White non-Hispanic or other/multiracial), BMI (<30 or ≥30), smoking status (ever or never smoker), and drinking status (ever or never drinker). Potential modifying effects were examined by testing the corresponding multiplicative interaction terms. All analyses were performed using the R version 4.3.1 software. The significance threshold was set at a *p*-value of <0.05 (two-sided).

## Results

### Participant characteristics

Among the 2,170 participants included in this study, 477 (22.0%) were diagnosed with T2DM (Table 1). The median dietary selenium intake was 106.4 μg/d, and participants with T2DM tended to be older, have higher BMI, and exhibit elevated levels of serum selenium and HOMA-IR.

**Table 1:** Baseline characteristics of participants based on the condition of T2DM in NHANES 2011–2016.

Characteristic	Overall *N* = 2,170 (100%)^1^	T2DM *N* = 477 (18%)^1^	Without T2DM *N* = 1,693 (82%)^1^	*p*-value^2^
Sex (%)				0.059
Female	1,066 (50%)	204 (44.03%)	862 (51.31%)	
Male	1,104 (50%)	273 (55.97%)	831 (48.69%)	
Age (years)				<0.001**
20–39 years	700 (31.83%)	35 (7.67%)	665 (37.15%)	
40–59 years	683 (35.01%)	142 (33.88%)	541 (35.26%)	
60+ years	787 (33.16%)	300 (58.45%)	487 (27.58%)	
Ethnicity (%)				0.3
White non-Hispanic	890 (67.64%)	173 (65.67%)	717 (68.07%)	
Black non-Hispanic	431 (9.57%)	114 (12.66%)	317 (8.89%)	
Mexican American	288 (7.98%)	78 (7.99%)	210 (7.98%)	
Other/multiracial	305 (8.06%)	52 (7.34%)	253 (8.21%)	
Other Hispanic	256 (6.76%)	60 (6.34%)	196 (6.85%)	
PIR	2.74 (1.33, 4.76)	2.41 (1.27, 3.93)	2.85 (1.39, 4.95)	0.021*
Educational attainment				<0.001**
Less than high school	471 (15.42%)	143 (18.83%)	328 (14.67%)	
High school graduate/GED	476 (20.73%)	120 (27.54%)	356 (19.23%)	
Some college or AA	655 (32.61%)	128 (33.78%)	527 (32.35%)	
College graduate or above	568 (31.24%)	86 (19.85%)	482 (33.75%)	
BMI				<0.001**
Underweight	46 (1.97%)	4 (0.88%)	42 (2.21%)	
Normal	611 (27.70%)	70 (12.04%)	541 (31.16%)	
Overweight	696 (33.88%)	149 (32.12%)	547 (34.27%)	
Obesity	797 (36.45%)	250 (54.95%)	547 (32.36%)	
Smoking status				0.001**
Current smoker	444 (20.18%)	81 (16.16%)	363 (21.07%)	
Former smoker	517 (25.40%)	163 (34.73%)	354 (23.34%)	
Never smoker	1,209 (54.42%)	233 (49.11%)	976 (55.59%)	
Drinking status				0.002**
1–5 drinks/month	1,034 (50.59%)	242 (54.20%)	792 (49.77%)	
5–10 drinks/month	163 (9.89%)	29 (4.72%)	134 (11.07%)	
10+ drinks/month	296 (18.41%)	45 (13.22%)	251 (19.60%)	
Non-drinker	535 (21.10%)	140 (27.86%)	395 (19.57%)	
Albumin (g/L)	43 (41, 45)	43 (40, 45)	43 (41, 46)	0.006**
Serum creatinine (umol/L)	74.26 (62.76, 86.63)	78.68 (64.53, 91.94)	74.26 (62.76, 84.86)	0.004**
PFG (mmol/L)	5.55 (5.22, 6.05)	7.22 (6.33, 8.96)	5.44 (5.16, 5.83)	<0.001**
Insulin (uU/mL)	9.81 (6.10, 15.20)	13.29 (8.95, 22.97)	8.87 (5.69, 14.12)	<0.001**
HbA1c (%)	5.50 (5.20, 5.80)	6.40 (5.90, 7.34)	5.40 (5.10, 5.60)	<0.001**
HOMA-IR	2.46 (1.47, 4.20)	4.63 (2.81, 8.23)	2.16 (1.36, 3.48)	<0.001**
Hypertension				<0.001**
Hypertension	937 (39.54%)	340 (70.62%)	597 (32.70%)	
Without hypertension	1,233 (60.46%)	137 (29.38%)	1,096 (67.30%)	
Dietary selenium intake (μg/d)	106.4 (78.8, 141.4)	99.5 (75.7, 137.4)	108.0 (80.9, 142.3)	0.12
Selenium supplement (μg/d)	97.2 (59.3, 136.2)	89.5 (55.1, 129.5)	97.9 (59.8, 137.0)	0.2
Total selenium intake (μg/d)	204.0 (147.2, 272.2)	186.4 (139.8, 260.6)	206.4 (150.3, 274.0)	0.15
Serum selenium (μg/L)	128.9 (119.0, 139.6)	132.8 (121.5, 143.1)	128.1 (118.4, 138.7)	0.001**

^1^median (IQR) for continuous; *n* (%) for categorical. ^2^Chi-squared test with Rao & Scott’s second-order correction; Wilcoxon rank-sum test for complex survey samples. *p < 0.05. **p < 0.01. *p* < 0.05 are shown in bold.

### Association between dietary selenium intake and T2DM risk

**Figure 2:**
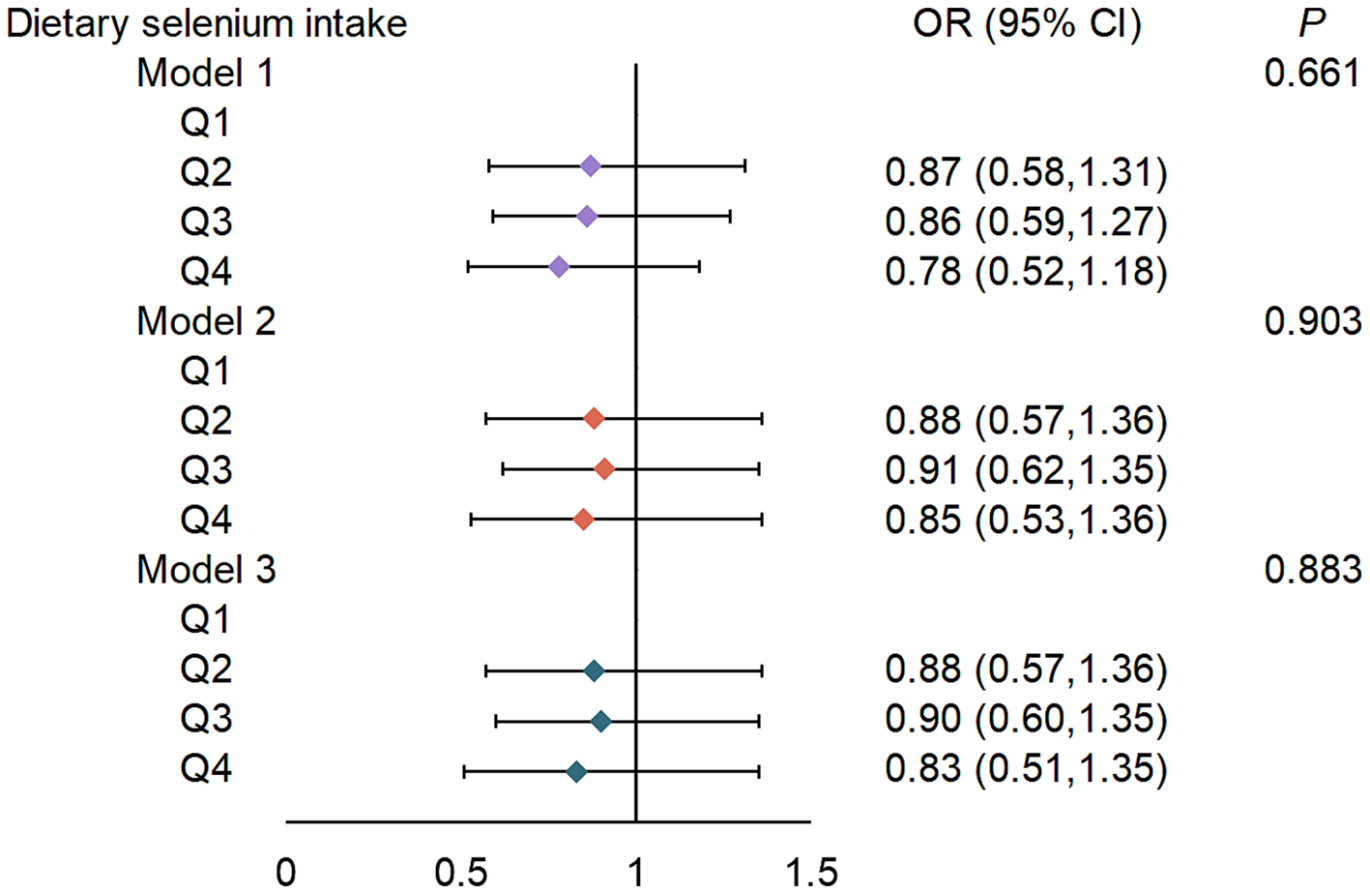
ORs (95% CIs) for the risk of T2DM according to dietary selenium intake. Q, quartile (μg/d). Q1, <78.85; Q2, 78.85–107.44; Q3, 107.45–142.05; Q4, >142.05. Model 1: adjusted for sex (male or female), age (continuous), ethnicity (White non-Hispanic, Black non-Hispanic, Mexican American, other/multiracial, and other Hispanic). Model 2: Model 1 + PIR (continuous), education level (less than high school, high school graduate/GED, some college or AA, or college graduate or above), BMI (continuous), smoking status (current smoker; former smoker, or never smoker), drinking status (1–5 drinks/month, 5–10 drinks/month, 10+ drinks/month, or non-drinker). Model 3: Model 2 + albumin (continuous), serum selenium (continuous), and serum creatinine (continuous).

**Figure 3:**
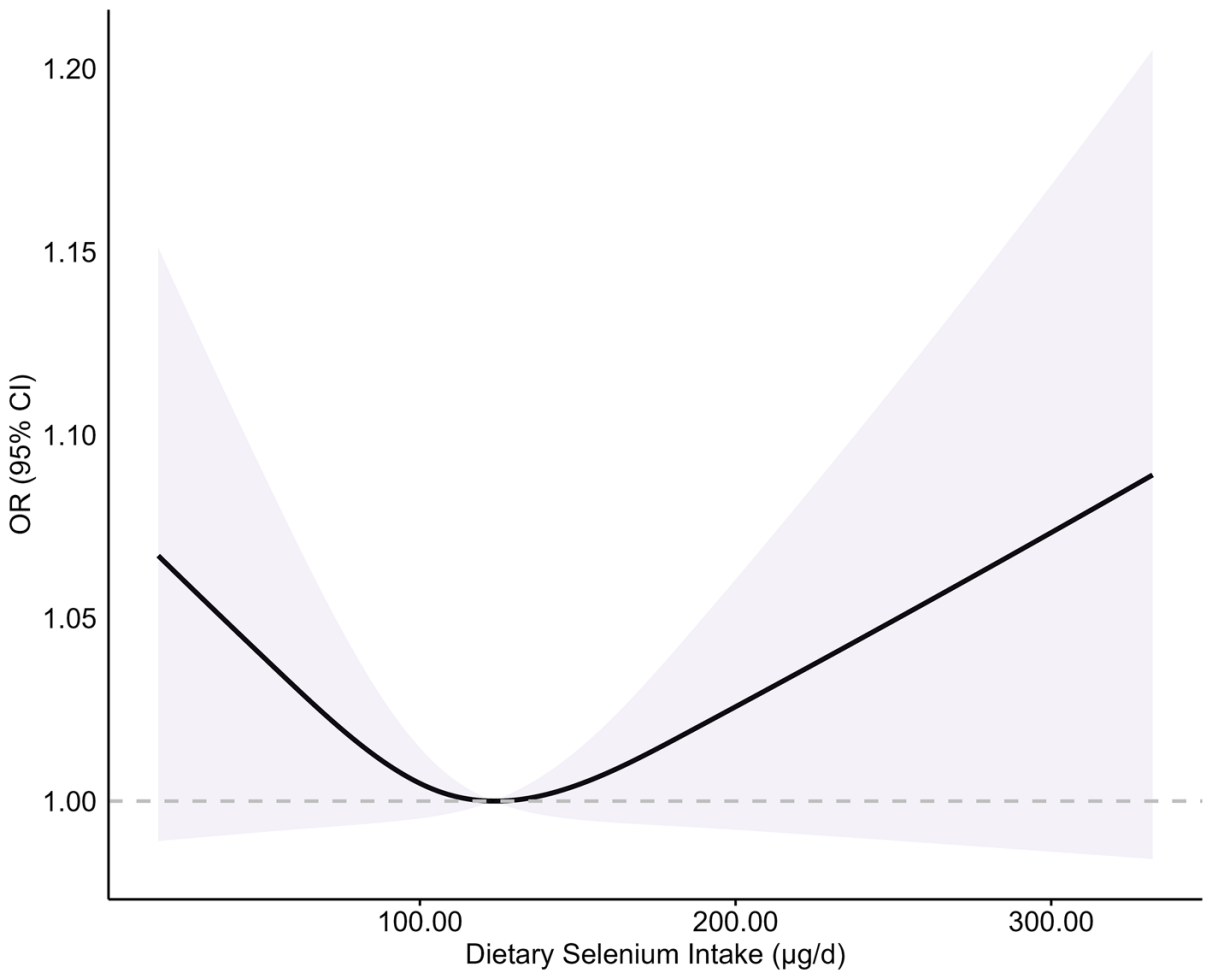
Association between dietary selenium intake and risk of T2DM. Adjusted for sex (male or female), age (continuous), ethnicity (White non-Hispanic, Black non-Hispanic, Mexican American, other/multiracial, and other Hispanic), PIR (continuous), education level (less than high school, high school graduate/GED, some college or AA, or college graduate or above), BMI (continuous), smoking status (current smoker; former smoker, or never smoker), drinking status (1–5 drinks/month, 5–10 drinks/month, 10 + drinks/month, or non-drinker), albumin (continuous), serum selenium (continuous), and serum creatinine (continuous). A dietary selenium intake level of 123.16 μg/d was used as the reference to estimate all ORs. The shaded areas indicate the 95% CI. *p*-non-linear = 0.042.

While quartile-based analysis showed non-significant linear trends (Q4 vs. Q1 OR = 0.83, 95% CI: 0.51–1.35) (Figure 2), spline regression revealed a significant U-shaped association between dietary selenium intake and T2DM risk (*p*-non-linear = 0.042) (Figure 3). This finding suggests that both low and high selenium intake levels are associated with increased T2DM risk. In contrast, no significant association with the risk of T2DM was observed in selenium supplements and total selenium intake (Supplementary Figure S1).

### Association between selenium intake and T2DM risk in subgroup analysis

Stratified analyses highlighted significant interactions in the obese subgroup (Table 2). Among participants with a BMI of ≥30, higher dietary selenium intake was associated with a reduced risk of T2DM (*p*_trend_ = 0.016). This association was not observed in non-obese participants, suggesting that selenium intake may play a protective role, specifically in populations with increased oxidative stress and metabolic demands. No significant interactions were detected between dietary selenium intake and strata variables (all *p*_interaction_ > 0.05).

**Table 2:** Stratified analyses of the associations (ORs, 95% CIs) between dietary selenium intake and the risk of T2DM^1^.

Characteristic	Dietary selenium intake (μg/d)				
	Q1 (<78.85)	Q2 (78.85–107.44)	Q3 (107.45–142.05)	Q4 (>142.05)	*p*_trend_
Sex					
Female	1.00	0.86 (0.48, 1.54)	1.07 (0.53, 2.15)	0.48 (0.16, 1.44)	0.272
Male	1.00	1.14 (0.44, 2.94)	0.79 (0.39, 1.61)	1.00 (0.46, 2.20)	0.746
Age (years)					
20–39	1.00	1.59 (0.55, 4.56)	1.13 (0.36, 3.54)	0.91 (0.26, 3.19)	0.758
40–59	1.00	0.85 (0.39, 2.13)	0.87 (0.36, 2.13)	1.42 (0.58, 3.49)	0.455
≥60	1.00	0.93 (0.47, 1.84)	0.95 (0.57, 1.57)	0.55 (0.27, 1.11)	0.758
Ethnicity (%)					
White non-Hispanic	1.00	0.91 (0.50, 1.66)	0.98 (0.57, 1.71)	0.84 (0.41, 1.69)	0.652
Other	1.00	1.10 (0.60, 2.01)	0.79 (0.46, 1.34)	0.90 (0.50, 1.59)	0.457
BMI (kg/m^2^)					
<30	1.00	0.90 (0.44, 1.87)	1.00 (0.44, 2.26)	1.46 (0.68, 3.14)	0.321
≥30	1.00	1.02 (0.52, 1.99)	0.75 (0.43, 1.30)	0.46 (0.23, 0.91)	0.016*
Smoking status					
Ever	1.00	0.72 (0.34, 1.51)	0.99 (0.57, 1.73)	1.13 (0.60, 2.14)	0.453
Never	1.00	1.25 (0.62, 2.53)	0.85 (0.41, 1.79)	0.70 (0.31, 1.59)	0.266
Drinking status					
Ever	1.00	0.90 (0.45, 1.78)	0.89 (0.52, 1.53)	0.88 (0.44, 1.75)	0.687
Non-drinker	1.00	0.92 (0.41, 2.06)	0.88 (0.42, 1.84)	0.64 (0.24, 1.70)	0.330

^1^Logistic regression models were used to estimate the ORs (95% CIs) of T2DM according to quartiles of dietary selenium intake. The results were adjusted for sex (male or female), age (continuous), ethnicity (White non-Hispanic, Black non-Hispanic, Mexican American, other/multiracial, or other Hispanic), PIR (continuous), an education level (less than high school, high school graduate/GED, some college or AA, or college graduate or above), BMI (continuous), smoking status (current smoker; former smoker, or never smoker), drinking status (1–5 drinks/month, 5–10 drinks/month, 10+ drinks/month, or non-drinker), albumin (continuous), serum selenium (continuous), and serum creatinine (continuous). The strata variable was not included in the model when stratifying by itself. PIR, poverty income ratio; BMI, body mass index. *p < 0.05. *p* < 0.05 are shown in bold.

## Discussion

This study revealed a significant U-shaped association between dietary selenium intake and the risk of T2DM using a restricted cubic spline model (*p*-non-linearity = 0.042). This finding offers a new perspective for explaining previous research contradictions: When the non-linear effect is ignored, the overall population may show a false zero association, while the risk heterogeneity at both ends of the threshold can reconcile the differences in results from different regional studies. For example, European populations generally have insufficient selenium intake due to low soil selenium content (serum selenium <90 μg/L). Randomized controlled studies supplementing selenium have found that increasing selenium intake can reduce glycation reactions and improve glucose metabolism indicators (13). In a randomized controlled study in Iran, selenium supplementation improved insulin resistance levels in patients with type 2 diabetes (14). Studies in high-selenium regions have found that high selenium intake increases the risk of diabetes (7–9). Geographical variations in soil selenium content and dietary intake likely shape this relationship, with selenium excess being harmful in replete populations and protective effects observed in deficient regions. Future studies should stratify analyses by baseline selenium levels and explore temporal trends to clarify compensatory responses versus causal mechanisms in diabetes progression.

The U-shaped relationship parallels the dual regulatory roles of selenoproteins in glucose homeostasis. At physiological levels, glutathione peroxidase 1 (GPX1) and thioredoxin reductase (TXNRD) neutralize reactive oxygen species (ROS), protecting β-cells and insulin-sensitive tissues (15–17). However, both deficiency and high levels of selenoproteins may promote T2DM (18). For instance, experiments in mice indicate that either knockout or overexpression of GPX1 may increase the risk of T2DM (19). Similarly, selenoprotein P (SELENOP), a hepatogenic secreted protein, may induce insulin resistance by inhibiting adenosine monophosphate-activated protein kinase (20). Clinical evidence further supports this duality: serum SELENOP levels increase during prediabetes but decline as T2DM progresses, suggesting compensatory overexpression preceding selenoprotein exhaustion (21). In addition, selenoprotein T, critical for endoplasmic reticulum proteostasis, governs insulin biosynthesis and secretion—a process vulnerable to selenium imbalance (22). These findings highlight the necessity of maintaining selenium within a narrow optimal range to prevent selenoprotein dysregulation.

Elevated serum selenium levels in T2DM patients may reflect a dynamic interplay between oxidative stress adaptation and selenoprotein dysregulation. Chronic hyperglycemia induces systemic oxidative stress, potentially triggering a compensatory upregulation of SELENOP to mobilize selenium in response to redox challenges (23). Although this response may transiently enhance antioxidant defenses, it paradoxically exacerbates insulin resistance by inhibiting AMPK signaling (24). Prolonged high selenium exposure can further impair β-cell function and insulin sensitivity through pro-oxidant selenium metabolites (5, 25).

Interestingly, we observed that higher selenium intake was associated with reduced T2DM risk exclusively in obese individuals. Obesity drives chronic oxidative stress through mitochondrial dysfunction and pro-inflammatory adipokine secretion, depleting antioxidant reserves (26–28). Selenium sufficiency supports selenoprotein-mediated detoxification of lipid peroxides and hydrogen peroxide, thereby preserving insulin signaling pathways (17). Preclinical studies corroborate this: selenium supplementation restores GPX1 activity in obese rodents, ameliorating insulin resistance and hepatic steatosis. However, Robert Hauffe et al. recently demonstrated that obesity blunts selenium’s insulin-sensitizing effects by dysregulating redox-sensitive phosphatases, highlighting the complex interplay between adiposity and selenium biology (29). These mechanistic insights align with our findings, suggesting that selenium-rich diets may benefit obese individuals with suboptimal selenium status.

Several limitations should be considered. First, due to its cross-sectional design, this study cannot establish a definitive causal relationship between selenium intake and the risk of T2DM. Residual reverse causation may persist, as undiagnosed prediabetic individuals might modify dietary habits, potentially attenuating observed associations. Second, the findings are based on data from U.S. adults, which may limit their generalizability to other populations. Third, geographical variations in soil selenium content may influence food-derived selenium levels, potentially affecting the accuracy of dietary selenium intake assessments. Fourth, self-reported diabetes diagnoses could lead to misclassification, particularly in undiagnosed cases. Finally, residual confounding by unmeasured factors cannot be excluded. Future research should integrate biomarkers and explore gene–environment interactions to refine precision nutrition strategies.

## Conclusion

In a nationally representative sample of U.S. adults, we found a U-shaped pattern between dietary selenium intake and the risk of T2DM. Among individuals with obesity, insufficient dietary selenium intake may contribute to the development of T2DM.

## Data Availability

The datasets presented in this study can be found in online repositories. The names of the repository/repositories and accession number(s) can be found at: http://www.cdc.gov/nchs/nhanes.htm.

## Supplementary material

SUPPLEMENTARY FIGURE S1 Associations between dietary selenium intake, selenium supplements, total selenium intake, and risk T2DM. **(A,D,G)** Dietary selenium intake; **(B,E,H)** selenium supplements; **(C,F,I)** total selenium intake. Model 1: Adjusted for sex (male or female), age (continuous), race (White non-Hispanic, Non-Hispanic Black, Mexican American, Other/multiracial, and Other Hispanic). Model 2: Model 1 + PIR (continuous), education level (less than high school, high school graduate/GED, some college or AA, and College graduate or above), BMI (continuous), smoking status (current smoker; former smoker, and never smoker), drinking status (1–5 drinks/month, 5–10 drinks/month, 10+ drinks/month, and non-drinker). Model 3: Model 2 + albumin (continuous), serum selenium (continuous), serum creatinine (continuous).
