# Supplementary figures and images for: A cross-sectional analysis of dietary selenium intake and type 2 diabetes risk in adults: insights from NHANES 2011–2016
Source: Front Nutr. 2025 May 9;12:1583590. doi: 10.3389/fnut.2025.1583590 (PMC12101123; doi:10.3389/fnut.2025.1583590)

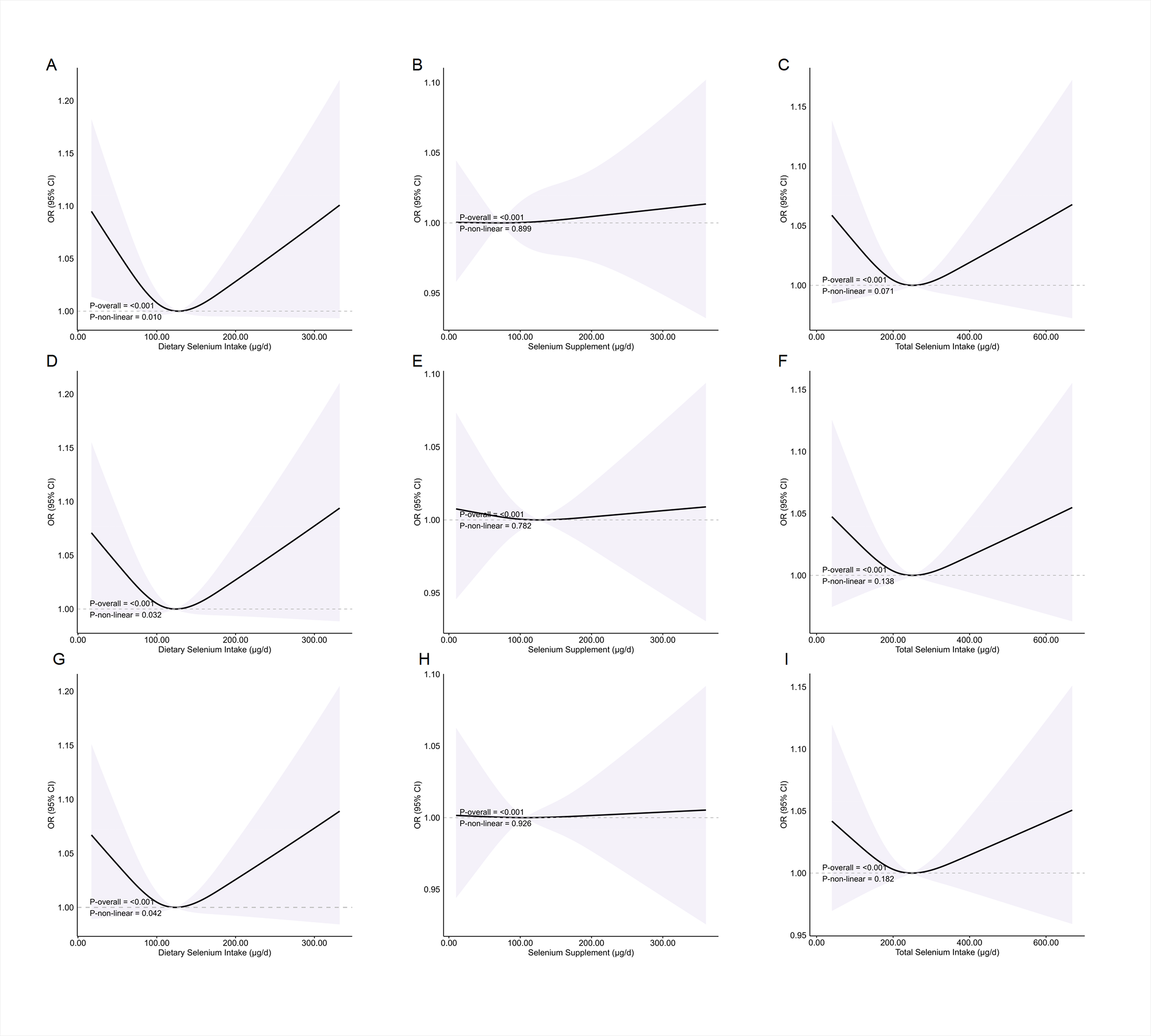

Supplement: SUPPLEMENTARY FIGURE S1 — Associations between dietary selenium intake, selenium supplements, total selenium intake, and risk T2DM. (A,D,G) Dietary selenium intake; (B,E,H) selenium supplements; (C,F,I) total selenium intake. Model 1: Adjusted for sex (male or female), age (continuous), race (White non-Hispanic, Non-Hispanic Black, Mexican American, Other/multiracial, and Other Hispanic). Model 2: Model 1 + PIR (continuous), education level (less than high school, high school graduate/GED, some college or AA, and College graduate or above), BMI (continuous), smoking status (current smoker; former smoker, and never smoker), drinking status (1–5 drinks/month, 5–10 drinks/month, 10+ drinks/month, and non-drinker). Model 3: Model 2 + albumin (continuous), serum selenium (continuous), serum creatinine (continuous). [file Image_1.TIF]
